# Accuracy and completeness of mortality data in the Department of Veterans Affairs

**DOI:** 10.1186/1478-7954-4-2

**Published:** 2006-04-10

**Authors:** Min-Woong Sohn, Noreen Arnold, Charles Maynard, Denise M Hynes

**Affiliations:** 1 Midwest Center for Health Services and Policy Research, 5^th ^Avenue and Roosevelt Road, Bldg 1, Room B259 (151H), Hines, IL 60141, USA; 2 Feinberg School of Medicine, Northwestern University, 676 N. St. Clair Suite 200, Chicago, IL 60611, USA; 3 Veterans Affairs Information Resource Center, 5^th ^Avenue and Roosevelt Road, Bldg 1, Room C305 (151V), Hines, IL 60141, USA; 4 Epidemiologic Research and Information Center, Department of Veterans Affairs Puget Sound Health Care System, 1660 S. Columbian Way, Seattle, WA 98108, USA; 5 Loyola University Chicago, Maywood, IL 60153, USA

## Abstract

**Background:**

One of the national mortality databases in the U.S. is the Beneficiary Identification and Record Locator Subsystem (BIRLS) Death File that contains death dates of those who have received any benefits from the Department of Veterans Affairs (VA). The completeness of this database was shown to vary widely from cohort to cohort in previous studies. Three other sources of death dates are available in the VA that can complement the BIRLS Death File. The objective of this study is to evaluate the completeness and accuracy of death dates in the four sources available in the VA and to examine whether these four sources can be combined into a database with improved completeness and accuracy.

**Methods:**

A random sample of 3,000 was drawn from 8.3 million veterans who received benefits from the VA between 1997 and 1999 and were alive on January 1, 1999 according to at least one source. Death dates found in BIRLS Death File, Medical SAS Inpatient Datasets, Medicare Vital Status, and Social Security Administration (SSA) Death Master File were compared with dates obtained from the National Death Index. A combined dataset from these sources was also compared with National Death Index dates.

**Results:**

Compared with the National Death Index, sensitivity (or the percentage of death dates correctly recorded in a source) was 77.4% for BIRLS Death File, 12.0% for Medical SAS Inpatient Datasets, 83.2% for Medicare Vital Status, and 92.1% for SSA Death Master File. Over 95% of death dates in these sources agreed exactly with dates from the National Death Index. Death dates in the combined dataset demonstrated 98.3% sensitivity and 97.6% exact agreement with dates from the National Death Index.

**Conclusion:**

The BIRLS Death File is not an adequate source of mortality data for the VA population due to incompleteness. When the four sources of mortality data are carefully combined, the resulting dataset can provide more timely data for death ascertainment than the National Death Index and has comparable accuracy and completeness.

## Background

Accurate data for mortality ascertainment are of critical importance for epidemiologic and health care outcomes studies. One of the national mortality databases in the U.S. is the Beneficiary Identification and Record Locator Subsystem (BIRLS) Death File that contains death dates of those who have received any benefits from the Department of Veterans Affairs (VA) since the early 1970s. This database has been widely used as the main source of death dates for veterans who received health care from the VA [1-5]. The completeness of this database has been shown to vary widely from cohort to cohort, ranging between 70.0% and 96.5% [6-12] in sensitivity or the percentage of death dates that are correctly recorded.

Three other databases for mortality ascertainment are available in the VA that can supplement the BIRLS Death File. One of them is the VA health care inpatient datasets. Another is the Social Security Administration (SSA) Death Master File which the VA acquires from the SSA. A third source is a subset of the Medicare Vital Status file that contains death dates for all Medicare-enrolled veterans. It is the newest source of mortality data for veterans which became available in the VA in 1999.

This study had two main objectives. The first was to evaluate the completeness and accuracy of death dates in these four sources of mortality data for a sample representative of the VA population. The second objective was to examine whether these four sources could be combined into a database with improved accuracy and completeness for mortality ascertainment.

## Methods

### Study population

This study was approved by the Human Subjects Committee and the Research and Development Committee at the Edward Hines Jr. VA Hospital in Hines, Illinois. The study population comprised all veterans who received any benefits from the VA between 1997 and 2002 and were alive on January 1, 1999 according to at least one source of mortality data available within the VA. Veterans who were enrolled in the Veterans Health Administration (VHA), received compensation or pension benefits, or utilized VA health care were included. After excluding those with no date of birth (20,049, 0.2%), those with invalid Social Security Numbers (10,940, 0.1%), and those who were Medicare beneficiaries but did not have an updated record in the Medicare Vital Status file (53,943, 0.6%), 8.3 million veterans were in the sampling frame.

### Mortality data available in the VA

The BIRLS is a VA database that contains information on all VA beneficiaries, including veterans discharged from military service since March 1973, Medal of Honor recipients, veterans who received education benefits from the VA, and veterans whose survivors applied for burial benefits [11]. This database contains death dates reported by family members applying for death benefits, VHA hospitals, or the VA National Cemetery Administration. The BIRLS Death File is a subset of the BIRLS that contains data on deceased veterans, including death dates.

The VA database called the Medical SAS Inpatient Datasets (MSID) is another source of death dates for veterans and contains information on patients who are discharged each year from any of the VHA hospitals across the county [13]. This database has been compiled each year since 1970 and includes dates of deaths that occurred in VHA hospitals or shortly after discharge [11]. It has previously been referred to as the Patient Treatment Files or PTF [14].

The Medicare Vital Status file is a dataset constructed by the Centers for Medicare and Medicaid Services (CMS) and contains demographic and vital status information for all Medicare beneficiaries. The VA has a data sharing agreement with the CMS to receive annually all Medicare data for VA-enrolled veterans [15]. The primary source for dates of death in the Vital Status file is the Death Master File compiled by the Social Security Administration (SSA), but the CMS also updates the Vital Status file with dates of death from other sources, including Medicare claims data [16].

The Death Master File is produced by the SSA, contains over 70 million deaths, and is updated monthly. This file is populated with death dates which SSA obtains from death reports by family members, funeral homes, state and federal agencies, postal authorities and financial institutions. Previous studies reported sensitivity for the SSA Death Master File ranging between 83% and 95% compared with the National Death Index [9,17-20]. The VA regularly obtains the SSA Death Master File and its monthly updates from the SSA, and makes them available to the researchers.

The data from the fifth source, namely the National Death Index, are considered the "gold standard" for mortality ascertainment. The National Death Index was established in 1981 by the National Center for Health Statistics to be a central repository of computerized death records for the entire U.S. population. It contains dates and causes of death from actual death records filed in state vital statistics offices since 1979 [21]. Deaths that occurred in a calendar year are added to the National Death Index annually, about 12 months after the end of the year. The lag time between the occurrence and the reporting of a death in the National Death Index may be anywhere between 12 to 24 months. The data in the National Death Index have been evaluated against known deaths from sources such as actual death certificates or direct contact with patients or their families, and have consistently exceeded 95% sensitivity [6,7,22-25]. The National Death Index was used in this study as the gold standard in evaluating completeness and accuracy of death dates from the four sources.

### National Death Index search

The study sample consisted of 3,000 veterans who were randomly drawn from the sampling frame. It was submitted to the National Death Index for a death date search in January, 2005. We provided Social Security Number (SSN), last name, first name, middle name, date of birth, date of death, sex, and state of residence.

The National Death Index search often returns multiple records as possible matches. The National Death Index uses nine different matching criteria to select possible matches, some of which do not require a match on Social Security Number [21]. A set of criteria must be developed to determine if any of the possible matches are the correct ones. A "true match" is established when the record returned from the National Death Index and the submitted record both belong to the same individual according to chosen criteria. A liberal criterion may increase sensitivity rates of death reporting, but can also increase the number of false positives and thus decrease specificity [6,7]. A careful choice of match criteria has important implications for the comparison [11].

Three match criteria were used to establish "true matches" in this study. The first criterion matched on SSN, sex, and two parts of the date of birth (day, month, or year). The probability that the match according to this criterion was correct was estimated to be 95% or higher [26]. Two other match criteria were: at least 7 digits of the SSN, date of birth, last name, first name, middle initial if provided by both sources, and sex; and, at least 7 digits of the SSN, date of death, last name, first name, middle initial if provided by both sources, and sex. These two criteria were used to match those cases with SSNs whose two digits were transposed. We established 96.2% of all true matches using the first criterion and only 3.8% using the other two.

### Determining the best death dates by combining mortality data sources

We developed an algorithm to determine the best source of death dates among our study sample. This algorithm used other stratified samples drawn from the pool of veterans who had death dates recorded in any of the four sources. These samples included veterans with death dates (1) that appeared in only one source (4.4% of the study population with a death date in any of the four sources), (2) that appeared in more than one source and agreed (88.1%), and (3) that appeared in more than one source but did not all agree (7.5%). These samples were submitted to the National Death Index along with the study sample. The results from this process of combining data sources are detailed elsewhere and are available upon request [16]. Results for the study sample are described below.

### Statistical analysis

For each source, an individual was placed into one of the four categories defined in Table 1, depending on how a veteran's mortality status from the source agreed with that from the National Death Index. False positives (B) are "misreported" deaths in the sense that they are not found in the National Death Index. True negatives (D) are "unreported" deaths; they are not reported in the source but are in the National Death Index.

**Table 1:** Definitions and Identification Methods of Four Groups for Assessing Completeness and Accuracy of Mortality Data

Groups	Definition and Identification Method
A. True Positives	All deceased individuals who were identified as deceased in a source. A valid death date was found in both the source and the NDI.
B. False Positives	All living individuals who were identified as deceased in a source. They have a death date in the source but not in the NDI.
C. False Negatives	All deceased individuals who were identified as alive in a source. They have a death date in the NDI but not in the source.
D. True Negatives	All living individuals who were identified as living in a source. They do not have a death date in the NDI nor in the source.

Using the number of individuals in each group in Table 1, four comparison statistics were computed for each source with the National Death Index data as the gold standard. Sensitivity indicates the per cent of deaths that were correctly recorded in a source, and was computed as the ratio of true positives to true positives plus false negatives [A/(A+C)]. Specificity refers to the per cent of individuals without a death date in the National Death Index who were not recorded as deceased in a source. It is the ratio of true negatives to true negatives plus false positives [D/(B+D)]. Positive predictive value refers to the probability that an individual who was identified as deceased in a source was actually deceased, and is computed as the ratio of true positives to true positives plus false positives [A/(A+B)]. Negative predictive value refers to the probability that an individual who was identified as not deceased in a source was actually not deceased, and is computed as the ratio of true negatives to true negatives plus false negatives [D/(C+D)].

We then computed sensitivity by VA health care use groups. Three groups were defined based on any VHA health care use in 1999–2002: inpatient users, outpatient users, and non-users. Inpatient and outpatient use groups are not mutually exclusive. Almost all inpatient users (99.2%) also used outpatient care during this period. The sensitivity rates were lower for the users of outpatient care only, but we chose to report these rates for all outpatient users to make comparison with previous studies easier [10,12].

Finally, we computed agreement rates at three different levels of precision: an exact date match, a match within two days, and a year and month match. The agreement rate indicates the proportion of death dates in a source that match at a given level of precision with dates in the National Death Index.

We only used the death dates from the National Death Index that fell between January 1, 1999 and December 31, 2002 to allow for at least 24-month time-lag in death reporting in the National Death Index.

## Results

Of the 3,000 records submitted, the National Death Index rejected two records due to incomplete demographic data and returned match results for 2,998 records. Of the veterans with returned possible match records, only 292 (9.7%) could be linked to the sample records as "true matches" using the three match criteria discussed above. After deleting two records rejected by the National Death Index, we had the final sample with 2,998 veterans and 292 deaths confirmed by the National Death Index.

Table 2 describes the study population and the sample used in this study. The study sample was similar to the overall population in all measured characteristics.

**Table 2:** Veteran Population and Study Sample by Selected Individual Characteristics, 1999 – 2002

	Population		Sample	
	
	N	Column %	N	Column %
Total	8,284,166	100.00	2,998	100.0
Age on Jan. 1, 1999				
< 65	4,896,608	59.1	1,762	58.8
65 or over	3,387,558	40.9	1,236	41.2
Sex				
Female	410,274	5.0	143	4.8
Male	7,873,892	95.0	2,855	95.2
VHA use*				
Inpatient	1,099,600	13.3	387	12.9
Outpatient	5,786,477	69.8	2,101	70.1
No use	2,490,427	30.1	894	29.8
Medicare enrolment				
Enrolled	4,641,193	56.0	1,705	56.9
Not enrolled	3,642,973	44.0	1,293	43.1

* Based on any use of VA inpatient or outpatient care in 1999 – 2002. The column per cents do not add up to 100% because almost all inpatient users (99.2%) also used outpatient care at least once during this period.

Table 3 shows comparisons of VA mortality data with National Death Index data by source. The SSA Death Master File identified the largest number of deaths of all four sources, followed by the Medicare Vital Status and BIRLS Death File with 272, 246, and 229 deaths, respectively. The Inpatient Datasets identified only 35 deaths.

**Table 3:** Comparison of VA Mortality Data with NDI Data by Source (N = 2,998)*

Data Source	Deceased in Source	Deceased in NDI		Sensitivity	Specificity	PPV	NPV
						
		Yes	No		(95% Confidence Interval)		
BIRLS-DF	Yes	226	3	77.4	99.9	98.7	97.6
	No	66	2,703	(72.2 – 82.1)	(99.7 – 100.0)	(96.2 – 99.7)	(97.0 – 98.2)
MSID	Yes	35	0	12.0	100.0	100.0	91.3
	No	257	2,706	(8.49 – 16.3)	(99.9 – 100.0)	(90.0 – 100.0)	(90.3 – 92.3)
MVS	Yes	243	3	83.2	99.9	98.8	98.2
	No	49	2,703	(78.4 – 87.3)	(99.7 – 100.0)	(96.5 – 99.7)	(97.7 – 98.7)
SSA-DMF	Yes	269	3	92.1	99.9	98.9	99.2
	No	23	2,703	(88.4 – 94.9)	(99.7 – 100.0)	(96.8 – 99.8)	(98.7 – 99.5)
Combined data	Yes	287	5	98.3	99.8	98.3	99.8
	No	5	2,701	(96.0 – 99.4)	(99.6 – 99.9)	(96.0 – 99.4)	(99.6 – 99.9)

* NDI indicates the National Death Index; BIRLS-DF, the Beneficiary Identification and Resource Locator Subsystem Death File; MSID, the VHA Medical SAS Inpatient Datasets; MVS, Medicare Vital Status; SSA-DMF, the Social Security Administration Death Master File; PPV, positive predictive value; NPV, negative predictive value.

The sensitivity rates were 77.4% for BIRLS Death File, 12.0% for Inpatient Datasets, 83.2% for Medicare Vital Status, and 92.1% for SSA Death Master File. The low rate for the BIRLS Death File was mainly due to unreported (66) rather than misreported (3) deaths. There were 49 and 23 unreported deaths in the Medicare Vital Status and SSA Death Master File, respectively, and 3 misreported deaths in both. When the sensitivity was computed only for Medicare beneficiaries (N = 1,705), the Vital Status file far exceeded any other single source with 99.2% sensitivity.

The vast majority of the veterans in the sample used VA outpatient care (70.1%), while only 12.9% used inpatient care, and 29.8% never used any health care from the VA (Table 4). The inpatient users had the highest sensitivity for both BIRLS Death File and SSA Death Master File with 86.3% and 94.5%, respectively. The sensitivity for outpatient users was 80.2% for the BIRLS Death File and 93.0% for the SSA Death Master File. Compared with either group of VHA users, non-users showed much worse sensitivity for both data sources: 72.1% for the BIRLS Death File and 90.4% for the SSA Death Master File. The difference in sensitivities between inpatients and outpatients was about 6% for the BIRLS Death File and 1.5% for the SSA Death Master File, while the difference between inpatients and non-users was 14.2% for the BIRLS Death File and 4.1% for the SSA Death Master File.

**Table 4:** Sensitivity Rates of Mortality Data by Selected Subgroups and Source for Veterans, 1999 – 2002

Subgroups	N (%)	Sensitivity (95% Confidence Interval)**		
		
		BIRLS-DF	SSA-DMF	Combined
VHA Healthcare Use*				
Inpatient Users	387 (12.9)	86.3 (76.2 – 93.2)	94.5 (86.6 – 98.5)	100.0 (95.1 – 100.0)
Outpatient Users	2,101 (70.1)	80.2 (73.8 – 85.7)	93.0 (88.4 – 96.2)	100.0 (98.0 – 100.0)
Non-Users	894 (29.8)	72.1 (62.5 – 80.5)	90.4 (83.0 – 95.3)	95.2 (89.1 – 98.4)
Age on Jan. 1, 1999				
< 65	1,762 (58.8)	67.6 (55.5 – 78.2)	91.5 (82.5 – 96.8)	95.8 (88.1 – 99.1)
65 or older	1,236 (41.2)	80.5 (74.7 – 85.5)	92.3 (88.0 – 95.5)	99.1 (96.8 – 99.9)

* Based on any health care use in the VHA in 1999 – 2002.

** BIRLS-DF indicates the Beneficiary Identification and Record Locator Subsystem Death File; SSA-DMF, the Social Security Administration Death Master File.

In contrast, death dates in these four sources agreed with those in the National Death Index at extremely high rates (Table 5). The death dates in the Inpatient Datasets all agreed exactly with the dates from the National Death Index. The BIRLS Death File had the second highest agreement (96.9%). Both the Medicare and SSA sources had slightly lower agreement rates than the BIRLS Death File with 95.5% and 95.6%, respectively.

**Table 5:** Agreement Rates of VA Mortality Data with NDI Dates by Source and Level of Precision*

Source	Number of Deaths	Death Dates in a Source that Agree with NDI Dates (%)		
		
		Exactly	Within 2 Days	In Year and Month
BIRLS-DF	229	222 (96.9)	224 (98.2)	225 (98.7)
MSID	35	35 (100.0)	35 (100.0)	35 (100.0)
MVS	246	235 (95.5)	239 (97.2)	244 (99.2)
SSA-DMF	272	260 (95.6)	265 (97.4)	270 (99.3)
Combined	292	285 (97.6)	288 (98.6)	291 (99.7)

* NDI indicates the National Death Index; BIRLS-DF, the Beneficiary Identification and Resource Locator Subsystem Death File; MSID, the VHA Medical SAS Inpatient Datasets; MVS, Medicare Vital Status; SSA-DMF, the Social Security Administration Death Master File.

There were 292 deaths identified in the combined data, with five unreported and five misreported deaths. The sensitivity of the combined data was 98.3%; the specificity, 99.8%; the positive predictive value, 98.3%; and the negative predictive value, 99.8% (Table 3). For the inpatient and outpatient users, the sensitivities of the combined data were both 100%, indicating that the combined data are as complete as the National Death Index for the VHA users. The rates of agreement with the National Death Index were 97.6%, 98.6%, and 99.7% for exact date match, match within 2 days, and year and month match, respectively.

Finally, we counted the number of death dates which each source contributed to the combined data (Table 6). The SSA Death Master File contributed the largest number of deaths (265 deaths, 90.8%), followed by the Medicare Vital Status with 241 (82.5%), and the BIRLS Death File with 221 deaths (75.7%). Each source contributed to the combined data dates that were found uniquely in that source. The SSA Death Master File contributed the largest number of unique dates with 13, accounting for about 4.5% of all dates in the combined data. 2.4% of all dates in the combined data were uniquely contributed by the BIRLS Death File, followed by the Medicare Vital Status (1.7%), and the Inpatient Datasets (0.7%).

**Table 6:** Source of Death Dates in the Combined Data

	Source*			
	
	BIRLS-DF	MSID	MVS	SSA-DMF
A. Death dates found in source	229	35	246	272
B. Death dates from source used in combined data	221	35	241	265
C. Death dates found only in source	7	2	5	13
D. % found only in source (C/A)	3.1%	5.7%	2.0%	4.8%
E. % of all dates in combined data found only in source (C/292)	2.4%	0.7%	1.7%	4.5%

* BIRLS-DF indicates the Beneficiary Identification and Resource Locator Subsystem Death File; MSID, the VHA Medical SAS Inpatient Datasets; MVS, Medicare Vital Status; SSA-DMF, the Social Security Administration Death Master File.

## Discussion

This study shows that the BIRLS Death File was extremely accurate but not complete. Of all 292 deaths reported in the National Death Index, 22.6% (66) were not reported and 1.3% (3) were incorrectly reported in the BIRLS Death File. These results suggest that if used alone, the BIRLS Death File is not an adequate source of mortality data for the overall VA population.

Of all four sources available within the VA, the SSA Death Master File was the most complete one for the VA population. If a researcher had to choose any one source for mortality ascertainment for veterans, we recommend the SSA Death Master File. It identified considerably more deaths with slightly more false positives than the BIRLS Death File. However, this finding is not consistent with a study by Page and colleagues [9] who reported lower sensitivity for the SSA Death Master File than for the BIRLS Death File. Previous studies found that the SSA Death Master File had large variability in completeness of death reporting by age [18,19], and we suspect that differences in age distributions may explain the inconsistency between the two studies [19].

This study showed the Medicare Vital Status file to be an important supplemental source of mortality information. It contained death dates for veterans that were not found in any other source available in the VA. For Medicare-enrolled veterans, this file was the most accurate and complete of all the single sources considered in this study. The high sensitivity and agreement rates suggest that it can be used alone as a source of mortality data for Medicare-enrolled veterans.

When available sources were combined, the resulting mortality data proved to be highly comparable to the National Death Index in both accuracy and completeness. For the VHA users, the combined data were 100% complete and 97.9% accurate to the date compared with the National Death Index.

There are some advantages in using the combined data over the National Death Index. While the four data sources described above are readily available free of charge to researchers affiliated with the VA, the National Death Index requires fees for data search that can be quite substantial when data for a large number of subjects are searched. Since both the BIRLS Death File and the SSA Death Master File are updated monthly, they can be used to obtain more recent death dates than the National Death Index.

One limitation of this study is that we examined death dates for a limited time frame (1999 – 2002), while death dates in the BIRLS Death File precede the 1970s and those in the SSA Death Master File precede the 1940s [11]. The accuracy and completeness of the combined data observed for this sample may not apply to deaths that occurred before 1999 and especially to those that occurred before the mid-1970s, since the completeness of both the BIRLS Death File and the SSA Death Master File was poor until mid-1970's [6,19]. These results may also not be used in studies which follow mortality of veterans using identifiers other than Social Security Numbers, since the completeness of mortality data for those with and without Social Security Numbers are known to be quite different [6,9].

## Conclusion

We found that a combined data set could provide highly accurate and complete data for mortality ascertainment and that hardly any improvement in accuracy or completeness could be achieved by acquiring death dates from the National Death Index. The combined data set can also provide more timely mortality data than the National Death Index whose time-lag for death reporting can be up to 24 months. Given the time- and resource-intensive nature of combining these four sources, a centralized database can be greatly beneficial to researchers by providing them with easy access to high-quality mortality data and thus improving the quality of research involving veteran mortality ascertainment.

## Abbreviations

BIRLS – Beneficiary Identification and Record Locator Subsystem

BIRLS-DF – BIRLS Death File

CMS – Centers for Medicare and Medicaid Services

MSID – Medical SAS Inpatient Datasets, formerly known as PTF

MVS – Medicare Vital Status

NDI – National Death Index

SSA – Social Security Administration

SSA-DMF – Social Security Administration Death Master File

SSN – Social Security Number

VA – Department of Veterans Affairs

VHA – Veterans Health Administration

## Competing interests

The author(s) declare that they have no competing interests.

## Authors' contributions

MWS oversaw the main project (SDR 03-157), participated in the design, statistical analysis of data, and drafted the manuscript.

NA was responsible for data collection, management, statistical analysis, and editing the manuscript.

CM participated in the design of the study, data analysis, and edited the manuscript.

DMH oversaw the 2^nd ^grant (SDR 98-004), participated in the design of the study and the interpretation of results, and edited the manuscript.
